# Acknowledgement to the Reviewers of GMS Zeitschrift für Medizinische Ausbildung

**DOI:** 10.3205/zma001000

**Published:** 2016-02-15

**Authors:** Martin R. Fischer, Götz Fabry

**Affiliations:** 1 GMS Zeitschrift für Medizinische Ausbildung, Schriftleitung, Erlangen, Deutschland; 2 Klinikum der Universität München, Institut für Didaktik und Ausbildungsforschung in der Medizin, München, Deutschland; 3 GMS Zeitschrift für Medizinische Ausbildung, Stellvertretender Schriftleiter, Erlangen, Deutschland; 4 Albert-Ludwig-Universität Freiburg, Medizinische Fakultät, Abteilung für Medizinische Psychologie und Soziologie, Freiburg, Deutschland

## Acknowledgement

We gratefully acknowledge the valuable contribution of the following peer reviewers, who supported our Journal in 2015. We are looking forward to continuing our collaboration!

## Reviewers in alphabetical order

- Olaf Ahlers, Berlin
- Matthias Angstwurm, München
- Sebastian Arlt, Berlin
- Katrin Balzer, Lübeck
- Daniel Bauer, München
- Johannes Bauer, München
- Nicola Bauer, Bochum
- Erika Baum, Marburg
- Jan Becker, Münster
- Stefan Beckers, Aachen
- Katrin Bekes, Wien
- Pascal Berberat, München
- Christoph Berendonk, Bern
- Mathias Berger, Freiburg i. Br.
- Sarah Berger, Heidelberg
- Mathias Bertram, Witten
- Silke Biller, Basel
- Mats Blohm, Heidelberg
- Martin Boeker, Freiburg i. Br.
- Klaus Böhme, Freiburg
- Markus Bolzer, München
- Hans Martin Bosse, Düsseldorf
- Claudia Bozzaro, Freiburg i. Br.
- Jan Breckwoldt, Zürich
- Beate Brem, Bern
- Georg Breuer, Erlangen
- Irene Brunk, Berlin
- Heinz Bruppacher, Zürich
- Andreas Burger, Bochum
- Rainer Büscher, Essen
- Jean-Francois Chenot, Greifswald
- Alexander Damanakis, Marburg
- Katja Anne Dannenberg, Berlin
- Renate Deinzer, Gießen
- Winand Dittrich, Essen
- Jörg Eberhard, Hannover
- Jan P. Ehlers, Witten
- Maren Ehrhardt, Hamburg
- Michael Ewers, Berlin
- Götz Fabry, Freiburg i. Br.
- Folkert Fehr, Sinsheim an der Elzenz
- Martin R. Fischer, München
- Volkhard Fischer, Hannover
- Walburga Freitag, Hannover
- Hendrik Friederichs, Münster
- Angelika Hiroko Fritz, Essen
- Jan Gärtner, Freiburg i. Br.
- Kirsten Gehlhar, Oldenburg
- Waltraud Georg, Berlin
- Andreas Gerber, Köln
- Marianne Giesler, Freiburg
- Adrian Goeldlin, Bern
- Matthäus Grasl, Wien
- Richard Gray, Fareham (UK)
- Christian Gruber, Hannover
- Markus Gulich, Ulm
- Martin Haag, Heilbronn
- Rainer Haak, Leipzig
- Petra Hahn, Freiburg
- Wolfgang Hampe, Hamburg
- Sigrid Harendza, Hamburg
- Wolf Hautz, Bern
- Jan Hense, Giessen
- Matthias Heue, Düsseldorf
- Johanna Christine Hissbach, Hamburg
- Matthias Hofer, Düsseldorf
- Angelika Hofhansl, Wien
- Anke Hollinderbäumer, Mainz
- Angelika Homberg, Bielefeld
- Thorsten Hornung, Bonn
- Peter Hucklenbroich, Münster
- Bert Huenges, Bochum
- Alfons Hugger, Düsseldorf
- Jürgen in der Schmitten, Düsseldorf
- Fabian Jacobs, München
- Jana Jünger, Heidelberg
- Ingo Just, Hannover
- Andreas Jüttemann, Halle
- Sylvia Kaap-Fröhlich, Zürich
- Jens Kaden, Mannheim
- Martina Kadmon, Oldenburg
- Gudrun Karsten, Kiel
- Sven Karstens, Trier
- Kathrin Klimke-Jung, Bochum
- Michael Knipper, Giessen
- Ansgar Köchel, Heidelberg
- Sascha Köpke, Lübeck
- Markus Krautter, Heidelberg
- Karl-Friedrich Krey, Greifswald
- Frank Krummenauer, Witten
- Mareike Landmann, Köln
- Christiane Luderer, Halle (Saale)
- Cornelia Mahler, Heidelberg
- Simone Manhal, Graz
- Georg Marckmann, München
- Jörg Marienhagen, Regensburg
- Gabriela Marx, Göttingen
- Maren März, Leipzig
- Lukas Peter Mileder, Graz
- Brigitte Mueller-Hilke, Rostock
- Elisabeth Narciß, Mannheim
- Marcus Neudert, Dresden
- Wilhelm Niebling, Freiburg i. Br.
- Christoph Nikendei, Heidelberg
- Michael Noll-Hussong, Ulm
- Zineb Miriam Nouns, Bern
- Falk R. Ochsendorf, Frankfurt/Main
- Wolfgang Öchsner, Ulm
- Eva-Maria Panfil, Zürich
- Friedrich Paulsen, Erlangen
- Bettina Pfleiderer, Münster
- Swetlana Philipp, Jena
- Reinhard Putz, München
- Sabine Ramspott, München
- Éva Rásky, Graz
- Tobias Raupach, Göttingen
- Gilbert Reibnegger, Graz
- Kathrin Reichel, Berlin
- Matthias Richter, Halle
- Bernt-Peter Robra, Magdeburg
- Marco Roos, Erlangen
- Thomas Rotthoff, Düsseldorf
- Michael Rufer, Zurich
- Stefan Rüttermann, Frankfurt
- Sabine Salloch, Bochum
- Thorsten Schäfer, Bochum
- Stefan Schauber, Oslo
- Theresa Scherer, Bern
- Jan Schildmann, Bochum
- Claudia Schlegel, Bern
- Anita Schmidt, Erlangen
- Tabea Schmidt-Ott, Oldenburg
- Michael Schmidts, Wien
- Kai Schnabel, Bern
- Michael Scholz, Erlangen
- Hanna Schröder, Aachen
- Jobst-Hendrik Schultz, Heidelberg
- Christian Schulz, Düsseldorf
- Sylvie Schuster, Basel
- Katrin Schüttpelz-Brauns, Mannheim
- Frank Seibert-Alves, Frankfurt
- Anne Simmenroth-Nayda, Göttingen
- Melanie Simon, Aachen
- Andreas Söhnel, Greifswald
- Beat Sottas, Bourguillon
- Anke Spura, Magdeburg
- Oliver Stadler, Vierkirchen
- Florian Steger, Halle
- Jost Steinhäuser, Lübeck
- Tina Stibane, Marburg
- Stephanie Stock, Köln
- Friederike Störkel, Münster
- Sylvère Störmann, München
- Ulrich Stößel, Freiburg
- Bernhard Strauß, Jena
- Irmgard Streitlein-Böhme, Freiburg
- Juerg Streuli, Zurich
- Michael Studnicka, Salzburg
- Christian Thrien, Köln
- Nils Thiessen, Bonn
- Olaf van dem Knesebeck, Hamburg
- Elisabeth van Gessel, Genf
- Nico Vonneilich, Hamburg
- Michaela Wagner-Menghin, Wien
- Ursula Walkenhorst, Osnabrück
- Hans-Jürgen Wenz, Kiel
- Birgit Wershofen, München
- Jürgen Westermann, Lübeck
- Michael Wicht, Köln
- Andreas Winkelmann, Neuruppin
- Ulrich Woermann, Bern
- Michaela Zupanic, Witten

